# Family-centred care in adult intensive care units: A scoping review protocol of secondary evidence with a focus on the nursing role

**DOI:** 10.1016/j.mex.2026.104142

**Published:** 2026-09-03

**Authors:** Gabriele Alberio, Stefano Terzoni, Maura Lusignani, Alberto Lucchini, Silvia Cilluffo, Rosario Caruso

**Affiliations:** a School of Medicine and Surgery, University of Milano-Bicocca, Monza, MB, 20900, Italy; b Department of Biomedical Sciences for Health, University of Milan, Milan, 20133, Italy; c General Adult and Pediatric Intensive Care Unit, Fondazione IRCCS San Gerardo dei Tintori, Monza, Italy; d Health Professions Research and Evidence Transfer Unit, IRCCS MultiMedica, Sesto San Giovanni, Italy

**Keywords:** Family-centred care, Intensive care units, Critical care nursing, Secondary evidence, Scoping review, Protocol

## Abstract

Admission to an adult intensive care unit (ICU) is a profoundly disruptive experience for both patients and their families. Family-Centred Care (FCC) is the predominant framework for addressing their needs, yet the secondary evidence base is heterogeneous and the specific nursing contribution remains insufficiently theorised. This protocol describes a scoping review designed to systematically map the secondary evidence on FCC in adult ICUs, with particular attention to the nursing role, the nurse–family relationship, and the instruments used to measure FCC-related constructs. Conducted in accordance with the JBI methodology and reported following the PRISMA extension for Scoping Reviews (PRISMA-ScR), the review will search six databases (PubMed, CINAHL, Embase, Cochrane, Web of Science, and Scopus) alongside grey literature, with no date restrictions and eligible sources in English and Italian. Only formal secondary sources (i.e., systematic, scoping, integrative, and narrative reviews, clinical practice guidelines, consensus statements, and position papers) will be included. Two independent reviewers will screen records and extract data using a pre-tested charting tool. The resulting higher-order map of conceptual frameworks, interventions, implementation barriers and facilitators, measurement instruments, and outcomes will clarify the state of existing syntheses and orient future critical care nursing research, practice, and implementation.

• Provides a structured, reproducible methodological framework (JBI methodology, PRISMA-ScR reporting) to systematically map the extensive and heterogeneous secondary evidence on Family-Centred Care (FCC) in adult ICUs.
• Explicitly targets the underexplored nursing role, mapping how the nurse–family relational dynamic is conceptualised and implemented across existing reviews and guidelines.
• Offers a comprehensive, pre-tested, multi-matrix data extraction tool tailored to synthesise diverse evidence types, including interventions, implementation barriers and facilitators, psychometric instruments, and outcomes.

## Graphical abstract

**Figure:**
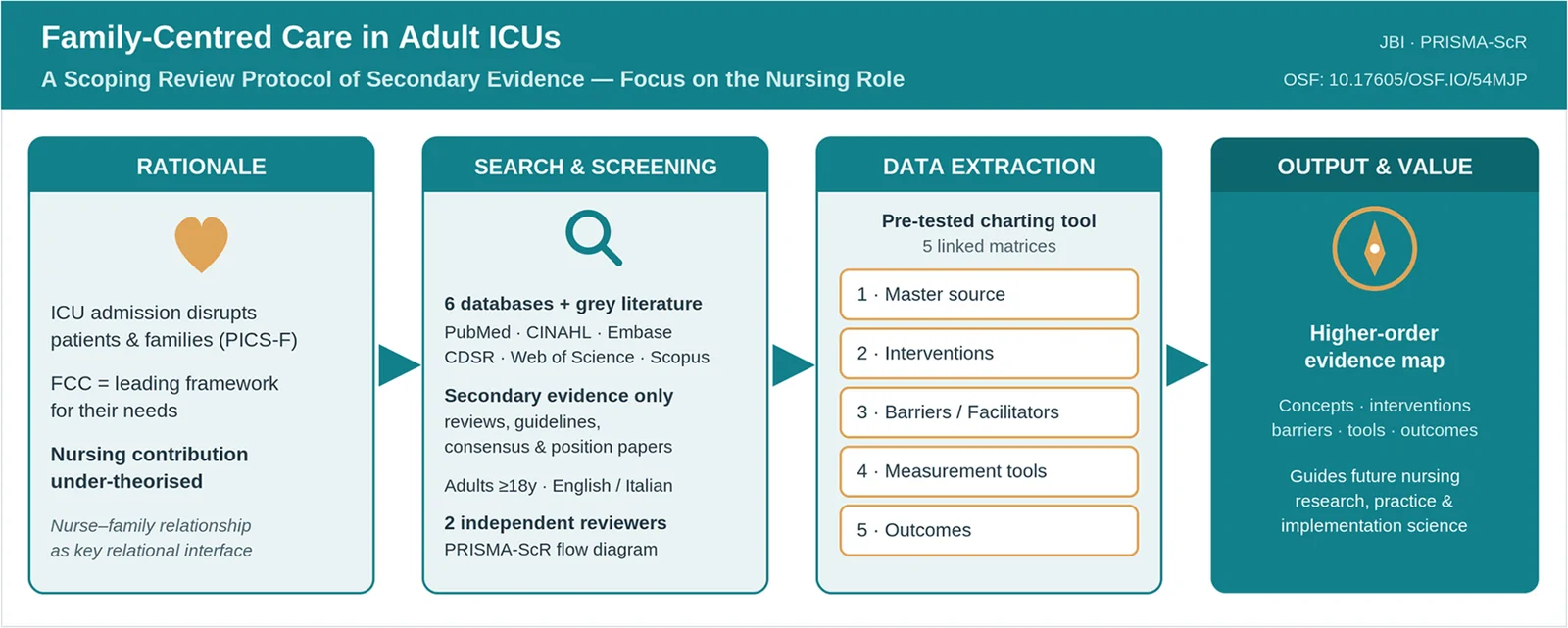

## Specifications table

**Table:** 

**Subject area**	Medicine and Dentistry
**More specific subject area**	Critical Care Nursing
**Name of your protocol**	Family-Centred Care in Adult Intensive Care Units: A Scoping Review Protocol of Secondary Evidence with a Focus on the Nursing Role
**Reagents/tools**	Not applicable
**Experimental design**	Scoping review protocol following the JBI methodological framework and PRISMA-ScR reporting guidelines to systematically map secondary evidence (systematic, scoping, integrative, narrative reviews, and clinical practice guidelines) on FCC in adult intensive care settings.
**Trial registration**	Registered on OSF Registries. Draft ID / Link: 10.17605/OSF.IO/54MJP
**Ethics**	Not applicable. This protocol describes a scoping review that synthesises previously published secondary evidence (reviews, clinical practice guidelines, consensus statements, and position papers). It does not involve human participants, animal experiments, or the collection of data from social media platforms. Therefore, informed consent, ethical approval, and the corresponding MethodsX statements on human subjects, animal experimentation, and social media data are not applicable.
Value of the Protocol	• Establishes a transparent, reproducible, and pre-planned methodology for mapping the extensive and highly heterogeneous body of secondary evidence on Family-Centred Care (FCC) in adult intensive care settings. • Focuses specifically on characterizing the nurse-family relational dynamic and the specific contribution of nursing staff within multidisciplinary critical care environments, which remains insufficiently theorized. • Systematically evaluates the psychometric properties and clinical applicability of measurement instruments used to assess FCC-related constructs, providing a higher-order map to guide future primary research and implementation science (EPIS framework).

## Background

Admission to an adult intensive care unit (ICU) is a profoundly disruptive experience for patients and families [1]. Family members face severe psychological distress, including anxiety, depression, and post-traumatic stress symptoms, described as Post-Intensive Care Syndrome–Family (PICS-F) [2]. The rigid, invasive nature of the ICU environment often disempowers families, relegating them to passive bystanders [3]. Family-Centred Care (FCC) has emerged as the leading framework to address these needs, encompassing open visiting, structured communication meetings, and family participation in care [4]. In 2017, the Society of Critical Care Medicine (SCCM) published comprehensive clinical guidelines for FCC in the neonatal, paediatric, and adult ICU [5]; many recommendations were graded as weak owing to methodological heterogeneity, underscoring the need to map existing knowledge before designing further evaluative trials [6].

Nurses occupy a structurally privileged bedside position to implement FCC, serving as the primary relational interface between families and the clinical team [6]. They mediate family presence, information sharing, and emotional support, yet the specific nursing contribution to FCC remains insufficiently theorised, often subsumed under broader multidisciplinary interventions [7]. As highlighted by the qualitative INpuT study, family involvement depends heavily on negotiated relational processes between nurses, patients, and families, necessitating explicit conceptual clarification [8]. Current syntheses also exhibit significant gaps: intervention descriptions are frequently inadequate, outcome measures are highly heterogeneous, and validated psychometric scales are inconsistently utilised [9]. Implementation is further restricted by multilevel structural and practical barriers, even when nursing staff maintain positive attitudes towards engagement [10,11].

Over the past two decades, a substantial volume of secondary literature has accumulated, including systematic, scoping, integrative, and narrative reviews, as well as clinical practice guidelines [1,[5], [6], [7]]. These sources differ fundamentally in inclusion criteria, conceptual definitions, and outcomes, making a higher-order synthesis necessary to clarify what has been synthesised and under what assumptions. To date, no scoping review has systematically mapped this secondary evidence with an explicit focus on the nursing role, the nurse–family relationship, and the measurement properties of FCC tools. Consolidating this fragmented evidence base into a single, structured map addresses a recognised gap at the intersection of FCC and critical care nursing, and gives researchers, clinicians, and guideline developers a shared reference point for how the nursing contribution to FCC has been conceptualised, implemented, and measured. For these reasons, a scoping review of secondary evidence is methodologically warranted to avoid duplication, identify gaps in existing syntheses, and orient future primary research and implementation science. This protocol aims to provide methodological guidance to systematically map the secondary evidence on Family-Centred Care in adult intensive care units, characterising how the nursing role and the nurse–family relationship are conceptualised, how FCC interventions are operationalised, what barriers and facilitators shape their implementation, which instruments are used to measure FCC-related constructs, and what patient, family, and provider outcomes have been reported.

## Description of protocol

### Protocol design and registration

This is a protocol for a scoping review of secondary evidence, conducted in accordance with the JBI methodology for scoping reviews [12] and reported following the PRISMA extension for Scoping Reviews (PRISMA-ScR) [13]. A scoping review design was chosen because the aim is to map the extent, range, and nature of an existing, heterogeneous secondary-evidence base, rather than to answer a narrowly defined effectiveness question or to produce a pooled quantitative estimate. The decision to map secondary rather than primary evidence is deliberate and central to the rationale. Over the past two decades, FCC in critical care has been the object of numerous systematic, scoping, integrative, and narrative reviews, as well as clinical practice guidelines and consensus statements [[2], [3], [4], [5], [6], [7],^9^,^10^]. This body of syntheses has grown to the point where it is itself fragmented and difficult to navigate: individual reviews adopt different conceptual definitions of FCC, apply divergent inclusion criteria, foreground different interventions and outcomes, and reach conclusions under differing methodological assumptions. When multiple overlapping syntheses coexist without a higher-order map, the resulting picture is redundant in some areas and silent in others, and it becomes unclear what has actually been synthesised, how, and with what gaps. Charting the secondary literature directly, i.e., an approach aligned with the logic of reviews of reviews/evidence overviews but performed coherently with the JBI guidance for scoping reviews [12], is therefore the most efficient and appropriate way to make this accumulated knowledge usable.

This approach is particularly valuable for three reasons. First, secondary sources are the level at which clinicians, educators, and guideline developers most often access evidence; clarifying their conceptual and methodological landscape has direct practical relevance for critical care nursing practice and policy. Second, mapping at the synthesis level avoids unnecessary duplication of primary-study screening and instead exposes meta-level gaps — constructs that remain undefined, populations that are under-represented, and measurement or implementation questions that no existing synthesis has adequately addressed. Third, by isolating how the nursing role and the nurse–family relationship are treated across existing syntheses, the review can identify where the specific nursing contribution has been rendered invisible within broader multidisciplinary framings, orienting future primary research, instrument development, and implementation science with precision.

The choice of a scoping review design, rather than an overview of reviews or an umbrella review, follows directly from the nature of the review questions [14]. Overviews and umbrella reviews are effectiveness-oriented designs: they typically restrict inclusion to systematic reviews (and meta-analyses), compare or re-aggregate their reported effect estimates to answer a focused question about the magnitude and certainty of an intervention's impact, and incorporate a formal appraisal of the methodological quality of the included reviews in order to weight or exclude them [15]. The present review has different objectives. It is deliberately concerned with the breadth and conceptual, methodological texture of the secondary evidence: how FCC and the nurse–family relationship are defined and theorised, how interventions are operationalised, which barriers and facilitators are described, which measurement instruments are used, and where the gaps lie. Answering these questions requires including a wider range of secondary source types, not only systematic reviews, but also scoping, integrative, and narrative reviews, clinical practice guidelines, consensus statements, and position papers, precisely the sources that an umbrella review would exclude but that carry much of the conceptual and normative content of the field [14,15]. It also requires mapping and charting characteristics rather than pooling results, and, consistent with JBI scoping review methodology, it does not use critical appraisal to exclude sources, even if critical appraisal could complement the scoping findings in an exploratory and descriptive way [12]. A scoping review of secondary evidence is therefore the design that matches the exploratory, mapping, and gap-identification aims of this study, whereas an umbrella review would answer a narrower effectiveness question over a narrower evidence base.

The protocol was registered on the Open Science Framework (OSF) Registries prior to formal screening [16]. Any amendments to the registered protocol will be documented, dated, and reported in the final review.

### Review questions

The review addresses one overarching question: What is the current state of secondary evidence on the conceptualisation, implementation, and outcomes of FCC in adult intensive care units, as reported in existing reviews, guidelines, and evidence syntheses, with particular attention to the nursing role and the nurse–family relationship?

This is operationalised through six sub-questions:

RQ1. What conceptual frameworks, theoretical models, and operational definitions have been used to describe FCC in adult ICU secondary literature, and how is the nurse–family relationship characterised within them?

RQ2. What is the scope, methodological diversity, and geographic distribution of existing secondary evidence on FCC in adult ICUs, and what gaps can be identified?

RQ3. What FCC interventions, programmes, and practice models implemented in adult ICUs have been described and synthesised, and how have they been operationalised?

RQ4. What barriers and facilitators to FCC implementation in adult ICUs have been reported, and at what levels (individual, organisational, systemic) do they operate?

RQ5. What instruments for assessing FCC-related constructs in adult ICUs have been reported, and what evidence do the sources provide on their psychometric properties and clinical applicability?

RQ6. What patient, family, and healthcare-provider outcomes associated with FCC in adult ICUs have been reported, and how have they been defined and measured?

### Eligibility criteria

Eligibility was defined using the JBI Population–Concept–Context (PCC) framework [12], complemented by an explicit specification of eligible source types and by a priori decision rules for borderline cases. To be included, a source must satisfy the PCC and Source-type criteria simultaneously; any case not resolved by the rules below is settled by consensus or a third reviewer (see Selection of sources).

**Population**. Because this review charts secondary literature, 'population' refers to the population addressed within each secondary source rather than to individually recruited participants. Eligible sources must focus explicitly on registered nurses (RNs) providing care in adult ICUs, on adult patients (aged ≥18 years) admitted to an ICU, or on family members and informal caregivers of adult ICU patients. In alignment with FCC principles, 'family' is defined as any person whom the patient — or, where the patient cannot communicate, the clinical record or surrogate — recognises as significant to their support, regardless of biological or legal ties. Sources addressing mixed professional populations (e.g., physicians, allied-health professionals, and nurses considered together) are eligible only if nursing-specific data are extractable, that is, if the source reports at least one nurse-attributable element chartable against the review questions, such as a nursing role or responsibility in FCC delivery, a nurse-led or nurse-mediated intervention component, nurses' attitudes or perceived barriers and facilitators, or an instrument administered to or completed by nurses; sources discussing FCC only at an aggregate team level, with no element attributable to nursing, are excluded. Where a source covers both adult and paediatric or neonatal populations, it is eligible only if adult-ICU data are reported separately or are otherwise extractable. Sources focused exclusively on non-nursing professionals, or exclusively on paediatric (PICU) or neonatal (NICU) settings, are excluded.

**Concept**. The central concept is Family-Centred Care in adult intensive care, in its conceptual, implementational, and evaluative dimensions. An eligible source must address at least one FCC dimension: conceptual models or definitions of FCC; the nurse–family relationship; the operationalisation of FCC interventions (e.g., flexible or open visiting, family presence during rounds or procedures, structured family communication and meetings, family participation in care, ICU diaries); barriers and facilitators to FCC implementation; instruments used to measure FCC-related constructs; or FCC-related outcomes for patients, families, or healthcare providers. Sources that mention 'family' only incidentally, without engaging any of these dimensions, are excluded.

**Context**. The context is restricted to adult ICUs or critical care units (CCUs) providing active intensive care, regardless of clinical specialty (medical, surgical, cardiac, neurological, trauma, or mixed). Sub-intensive and step-down or high-dependency units, general wards, emergency departments, and paediatric or neonatal units are excluded. Because family communication around end-of-life and treatment-limitation decisions is an integral component of FCC in active intensive care, a source is not excluded merely for addressing end-of-life or palliative content; it is excluded only when its stated scope or objective is exclusively palliative, hospice, or end-of-life/withdrawal-of-treatment care for patients with no prospect of recovery, or exclusively resuscitation manoeuvres, such that FCC in the context of ongoing active intensive care is not addressed.

**Types of sources**. Only formal secondary literature is eligible, comprising two groups. The first is evidence syntheses published in peer-reviewed journals [i.e., systematic reviews (with or without meta-analysis), scoping reviews, integrative reviews, narrative or literature reviews, qualitative evidence syntheses and meta-syntheses, rapid reviews, evidence and mapping reviews, and umbrella reviews or overviews of reviews] a review being eligible regardless of the label used by its authors, provided it constitutes a synthesis of previously published literature. The second is evidence-based clinical practice guidelines, consensus statements, and position papers issued or formally endorsed by a recognised professional body, defined as a national or international scientific or professional society, a critical-care or nursing association, or a governmental or health-technology-assessment (HTA) agency; for these document types, journal peer review is not required, but the issuing or endorsing body must be identifiable.

Excluded are primary studies (including primary studies embedded within, but separable from, an eligible review), study or review protocols, conference abstracts and proceedings, editorials, commentaries, letters and opinion pieces without a synthesis or endorsing body, theses and dissertations, textbooks, and non-endorsed web content. No date restrictions are applied in order to capture the full historical development of FCC secondary evidence, and eligible languages are English and Italian, reflecting the working languages of the review team; the potential for language bias is acknowledged as a limitation.

### Step-by-step procedure

#### Step 1 — information sources

Six bibliographic databases are searched: PubMed, CINAHL (EBSCOhost), Embase (Elsevier), the Cochrane Database of Systematic Reviews (CDSR, Cochrane Library), Web of Science (Core Collection), and Scopus. These are complemented by grey literature and reference-list screening (Steps 3–4).

#### Step 2 — search strategy

A three-phase approach is adopted. Phase 1 (completed) was a preliminary search in PubMed and CINAHL to identify controlled vocabulary and free-text terms. Phase 2 is the full search across the six databases. For five databases the strategy combines four conceptual blocks (Context AND Concept AND Population AND Source Type) via Boolean operators; for the CDSR, which by definition indexes only systematic reviews, the Source Type block is omitted and three blocks are combined. The complete, field-tested strings, execution dates, and records retrieved are reported in Table 1. Phase 3 is reference-list screening ('pearl growing') of all included sources.

**Table 1:** Field-tested search strings, execution dates, and records retrieved across the six databases.

**Database (interface)**	**Date executed**	**Search string**	**Records**
PubMed (NLM)	11/04/2026	("Intensive Care Units"[MeSH] OR "Critical Care"[MeSH] OR "Critical Care Nursing"[MeSH] OR "intensive care unit*"[Title/Abstract] OR "critical care unit*"[Title/Abstract] OR "ICU"[Title/Abstract] OR "CCU"[Title/Abstract] OR "intensive care"[Title/Abstract] OR "critical care"[Title/Abstract]) AND ("Family Nursing"[MeSH] OR "Professional-Family Relations"[MeSH] OR "Patient-Centered Care"[MeSH] OR "Family-Centered Care"[Title/Abstract] OR "Family-Centred Care"[Title/Abstract] OR "Patient- and Family-Centered Care"[Title/Abstract] OR "Family Involvement"[Title/Abstract] OR "Family Engagement"[Title/Abstract] OR "Family Presence"[Title/Abstract] OR "Family Participation"[Title/Abstract] OR "Open Visitation"[Title/Abstract] OR "Flexible Visiting"[Title/Abstract] OR "Visiting Polic*"[Title/Abstract] OR "Family Support"[Title/Abstract] OR "Nurse-Family Relation*"[Title/Abstract] OR "Nurse-Family Partnership"[Title/Abstract]) AND ("Nursing"[MeSH] OR "Nurses"[MeSH] OR "nurse*"[Title/Abstract] OR "nursing staff"[Title/Abstract] OR "registered nurse*"[Title/Abstract]) AND("systematic review"[Publication Type] OR "meta-analysis"[Publication Type] OR "practice guideline"[Publication Type] OR "guideline"[Publication Type] OR "systematic review"[Title/Abstract] OR "scoping review"[Title/Abstract] OR "integrative review"[Title/Abstract] OR "narrative review"[Title/Abstract] OR "evidence synthesis"[Title/Abstract] OR "literature review"[Title/Abstract] OR "clinical practice guideline"[Title/Abstract] OR "evidence-based guideline"[Title/Abstract])	129
CINAHL (EBSCOhost)	11/04/2026	((MH "Intensive Care Units+") OR (MH "Critical Care") OR (MH "Critical Care Nursing") OR TI "intensive care unit*" OR AB "intensive care unit*" OR TI "critical care unit*" OR AB "critical care unit*" OR TI ICU OR AB ICU OR TI CCU OR AB CCU OR TI "intensive care" OR AB "intensive care" OR TI "critical care" OR AB "critical care") AND ((MH "Family-Centered Care") OR (MH "Professional-Family Relations") OR (MH "Family Nursing") OR TI "family-centered care" OR AB "family-centered care" OR TI "family-centred care" OR AB "family-centred care" OR TI "patient- and family-centered care" OR AB "patient- and family-centered care" OR TI "family involvement" OR AB "family involvement" OR TI "family engagement" OR AB "family engagement" OR TI "family presence" OR AB "family presence" OR TI "family participation" OR AB "family participation" OR TI "open visitation" OR AB "open visitation" OR TI "flexible visiting" OR AB "flexible visiting" OR TI "visiting polic*" OR AB "visiting polic*" OR TI "family support" OR AB "family support" OR TI "nurse-family relation*" OR AB "nurse-family relation*" OR TI "nurse-family partnership" OR AB "nurse-family partnership") AND ((MH "Nurses+") OR (MH "Nursing Practice") OR TI nurse* OR AB nurse* OR TI "nursing staff" OR AB "nursing staff" OR TI "registered nurse*" OR AB "registered nurse*") AND ((PT "Systematic Review") OR (PT "Meta Analysis") OR (PT "Literature Review") OR (PT "Practice Guidelines") OR TI "scoping review" OR AB "scoping review" OR TI "integrative review" OR AB "integrative review" OR TI "narrative review" OR AB "narrative review" OR TI "evidence synthesis" OR AB "evidence synthesis" OR TI "clinical practice guideline" OR AB "clinical practice guideline")	120
Embase (Elsevier)	11/04/2026	('intensive care unit'/exp OR 'critical care'/exp OR 'critical care nursing'/exp OR 'intensive care unit*':ti,ab OR 'critical care unit*':ti,ab OR 'ICU':ti,ab OR 'CCU':ti,ab OR 'intensive care':ti,ab OR 'critical care':ti,ab) AND ('family centered care'/exp OR 'nurse patient relationship'/exp OR 'family nursing'/exp OR 'family-centered care':ti,ab OR 'family-centred care':ti,ab OR 'patient- and family-centered care':ti,ab OR 'family involvement':ti,ab OR 'family engagement':ti,ab OR 'family presence':ti,ab OR 'family participation':ti,ab OR 'open visitation':ti,ab OR 'flexible visiting':ti,ab OR 'visiting polic*':ti,ab OR 'family support':ti,ab OR 'nurse-family relation*':ti,ab OR 'nurse-family partnership':ti,ab) AND ('nurse'/exp OR 'nursing'/exp OR 'nurse*':ti,ab OR 'nursing staff':ti,ab OR 'registered nurse*':ti,ab) AND('systematic review'/exp OR 'meta analysis'/exp OR 'practice guideline'/exp OR 'systematic review':ti,ab OR 'scoping review':ti,ab OR 'integrative review':ti,ab OR 'narrative review':ti,ab OR 'evidence synthesis':ti,ab OR 'clinical practice guideline':ti,ab OR 'literature review':ti,ab)	377
CDSR – Cochrane Database of Systematic Reviews (Cochrane Library, Search Manager)	11/04/2026	#1 MeSH descriptor: [Intensive Care Units] explode all trees #2 MeSH descriptor: [Critical Care] explode all trees #3 MeSH descriptor: [Critical Care Nursing] explode all trees#4 ("intensive care unit*" OR "critical care unit*" OR "ICU" OR "CCU" OR "intensive care" OR "critical care"):ti,ab,kw #5 #1 OR #2 OR #3 OR #4 #6 MeSH descriptor: [Patient-Centered Care] explode all trees #7 MeSH descriptor: [Professional-Family Relations] explode all trees #8 MeSH descriptor: [Family Nursing] explode all trees #9 ("family-centered care" OR "family-centred care" OR "patient- and family-centered care" OR "family involvement" OR "family engagement" OR "family presence" OR "family participation" OR "open visitation" OR "flexible visiting" OR "visiting polic*" OR "family support" OR "nurse-family relation*" OR "nurse-family partnership"):ti,ab,kw #10 #6 OR #7 OR #8 OR #9 #11 MeSH descriptor: [Nurses] explode all trees #12 MeSH descriptor: [Nursing] explode all trees#13 ("nurse*" OR "nursing staff" OR "registered nurse*"):ti,ab,kw #14 #11 OR #12 OR #13 #15 #5 AND #10 AND #14 [results limited to Cochrane Reviews / CDSR]	0
Web of Science (Core Collection)	16/04/2026	TS=("intensive care unit*" OR "critical care unit*" OR "ICU" OR "CCU" OR "intensive care" OR "critical care") AND TS=("family-centered care" OR "family-centred care" OR "patient- and family-centered care" OR "family involvement" OR "family engagement" OR "family presence" OR "family participation" OR "open visitation" OR "flexible visiting" OR "visiting policy" OR "visiting policies" OR "family support" OR "nurse-family relationship*" OR "nurse-family partnership") AND TS=("nurse*" OR "nursing staff" OR "registered nurse*") AND (DT=(Review) OR TS=("systematic review" OR "scoping review" OR "integrative review" OR "narrative review" OR "clinical practice guideline" OR "evidence synthesis"))	115
Scopus (Elsevier)	11/04/2026	TITLE-ABS-KEY ("intensive care unit*" OR "critical care unit*" OR "ICU" OR "intensive care" OR "critical care") AND TITLE-ABS-KEY ("family-centered care" OR "family-centred care" OR "family involvement" OR "family engagement" OR "family presence" OR "family participation" OR "open visitation" OR "family support" OR "nurse-family relation*" OR "nurse-family partnership") AND TITLE-ABS-KEY ("nurse*" OR "nursing staff" OR "registered nurse*") AND(DOCTYPE(re) OR TITLE-ABS-KEY ("systematic review" OR "scoping review" OR "integrative review" OR "narrative review" OR "clinical practice guideline" OR "evidence synthesis"))	168
**Total records retrieved**			**909**

#### Step 3 — grey literature

Targeted manual searching is performed of the official archives of the Society of Critical Care Medicine (SCCM), the European federation of Critical Care Nursing associations (EfCCNa), the American Association of Critical-Care Nurses (AACN), the World Federation of Critical Care Nurses (WFCCN), and the Federazione Nazionale Ordini Professioni Infermieristiche (FNOPI). Google Scholar is screened for the first 10 pages of results, using a simplified string: ("intensive care" OR "critical care") AND ("family-centered care" OR "visiting policy") AND ("nurse" OR "nursing").

#### Step 4 — de-duplication and record management

All retrieved records are imported into a reference-management/systematic-review platform, where duplicates are removed prior to screening. The number of duplicates removed is recorded for the PRISMA-ScR flow diagram.

#### Step 5 — pilot testing

Before formal screening, the two reviewers independently apply the eligibility criteria to a random sample of 5% of the total records to calibrate interpretation and refine decision rules.

#### Step 6 — selection of sources (two-stage screening)

Screening proceeds in two independent stages: (1) title/abstract screening by two independent reviewers, and (2) full-text evaluation against the eligibility criteria by the same two reviewers. Disagreements at either stage are resolved by consensus or, if needed, by a third reviewer. Reasons for exclusion at full text are recorded and reported in the PRISMA-ScR flow diagram.

#### Step 7 — data charting (extraction)

Data are charted independently by two reviewers using a pre-tested, standardised charting tool comprising five linked matrices (see **Appendices 1–5**): (1) Master Source Extraction Form; (2) Intervention/Practice-Model Form; (3) Barriers and Facilitators Form; (4) Measurement-Instrument Form; and (5) Outcome Form. Each matrix is linked to the master record via a unique source ID, allowing multi-level indexing of the evidence base. The tool is piloted on three included sources to resolve operational ambiguities and maximise inter-rater agreement; charting is iterative, and the tool may be refined during the process, with any changes reported.

#### Step 8 — data analysis and presentation

Consistent with scoping review methodology, results are synthesised descriptively rather than pooled statistically. Charted data are summarised using (a) basic numerical/descriptive counts (e.g., source types, countries, publication years, intervention categories, instruments), presented in tables and figures; and (b) a narrative synthesis organised around the six review questions, mapping conceptual frameworks, interventions, barriers/facilitators, measurement instruments, and outcomes. Findings are presented against the RQ structure and accompanied by an explicit identification of gaps in the existing secondary evidence to orient future primary research and implementation science.

### Study guidelines and ethical statement

The review is conducted according to JBI scoping review methodology [12] and reported according to PRISMA-ScR [13]; a completed PRISMA-ScR checklist accompanies the final review. Consistent with scoping review methodology, a formal critical appraisal of the methodological quality of included sources is not performed to score or exclude them; however, whether each source itself conducted an independent critical appraisal is charted (**Appendix 1**). Ethical approval and informed consent are not required, as the review synthesises previously published secondary evidence and does not involve human participants, animal experimentation, or data collected from social media platforms. Guidelines specific to primary human-subjects research and to regulated interventional studies (e.g., FDA guidance) are not applicable to this design.

### Protocol validation

Results and discussion are not part of a protocol article, and, because this is a protocol for a scoping review of secondary evidence, traditional primary experimental validation data are not applicable. Nevertheless, the internal validity and feasibility of the protocol were verified before submission through four complementary procedures.

#### Overlap assessment against existing registrations

On 11/04/2026, the OSF Registries, the JBI systematic review register, and PROSPERO were checked for ongoing or completed reviews with an overlapping scope. Because PROSPERO does not register scoping reviews, it was consulted only to identify related systematic reviews that might duplicate the intended mapping; no scoping review of secondary evidence on FCC in adult ICUs with an explicit nursing focus was identified. The protocol was subsequently registered on OSF Registries [16] prior to formal screening, ensuring transparency and reducing the risk of unplanned duplication.

Full execution of the search strategy. The four-block strategy was translated into the native syntax of each database and executed in full across all six sources (see Table 1), confirming that every string compiles and runs without syntactic error and retrieves a tractable, topically relevant volume of records. Phase 2 yielded 909 records in total (PubMed 129; CINAHL 120; Embase 377; Web of Science 115; Scopus 168). As anticipated, the CDSR returned no unique records, consistent with the indexing of Cochrane systematic reviews in PubMed and Embase; the search was nonetheless executed and documented for reproducibility.

#### Sensitivity check against a seed set of known reviews

To verify that the strategy captures the relevant literature rather than merely returning a manageable number of records, its recall was checked against a predefined set of key secondary sources known to the review team a priori (e.g., [1,4,9]). All seed records were retrieved by the combined strings, supporting the adequacy of the conceptual blocks.

Planned calibration of screening and charting. Before the comprehensive screening and extraction phases begin, two operational pilots will be conducted to maximise inter-rater agreement. First, the two reviewers will independently apply the eligibility criteria to a random sample of 5% of the de-duplicated records, refining the decision rules where disagreements arise. Second, the five-matrix charting tool (**Appendices 1–5**) will be piloted on three included secondary sources to resolve operational ambiguities before full extraction. Inter-rater agreement at the pilot stage may be quantified (e.g., percentage agreement or Cohen’s κ) and reported in the final review.

### Limitations

This scoping review protocol has several limitations that should be acknowledged. First, eligibility is restricted to secondary sources published in English or Italian. Although this reflects the working languages of the review team, it introduces a potential language bias and may exclude relevant evidence syntheses, consensus statements, or clinical practice guidelines published in other languages, particularly those issued by non-Anglophone European critical-care and nursing bodies.

Second, by design the review maps only secondary evidence (reviews, guidelines, consensus statements, and position papers) and does not chart primary studies directly. This introduces an inherent temporal lag: primary studies published shortly before the search date that have not yet been incorporated into any synthesis will not be captured, and areas of active but not-yet-synthesised research may appear as gaps. The findings therefore describe the state of synthesised knowledge rather than the full primary evidence base.

Third, the Population block of the search strategy requires an explicit nursing term, which favours specificity over sensitivity. Secondary sources that contain extractable nursing-relevant content but frame it exclusively within an aggregate multidisciplinary vocabulary, without any nurse-specific term in the title, abstract, or indexing, may not be retrieved. This trade-off is mitigated, but not eliminated, by reference-list screening (‘pearl growing’), grey-literature searching, and the seed-set sensitivity check; residual under-retrieval of nursing content embedded in multidisciplinary syntheses remains possible.

Fourth, in accordance with scoping review methodology, no formal critical appraisal of the methodological quality of the included sources is undertaken to score or exclude them. Consequently, the review maps what has been synthesised and under what assumptions, but does not adjudicate the reliability of individual syntheses; to partially offset this, whether each source itself conducted an independent critical appraisal is charted (**Appendix 1**).

Finally, because family communication in the context of end-of-life and treatment-limitation decisions overlaps substantially with FCC, the decision to exclude sources focused exclusively on palliative or end-of-life care may omit some family-focused content situated at the boundary between active intensive care and end-of-life care; this boundary is applied conservatively and consistently across screening.

## CRediT authorship contribution statement

**Gabriele Alberio:** Conceptualization, Methodology, Investigation, Data curation, Writing – original draft, Project administration. **Stefano Terzoni:** Methodology, Validation, Writing – review & editing. **Maura Lusignani:** Methodology, Supervision, Writing – review & editing. **Alberto Lucchini:** Conceptualization, Validation, Writing – review & editing. **Silvia Cilluffo:** Methodology, Investigation, Data curation, Writing – review & editing. **Rosario Caruso:** Conceptualization, Supervision, Validation, Writing – review & editing.

## Declaration of competing interest

The authors declare that they have no known competing financial interests or personal relationships that could have appeared to influence the work reported in this paper.

## Data Availability

As this is a methodological protocol, no primary datasets were generated or analysed. The data extraction tools developed for the future scoping review are provided in full as Supplementary Material.

## Appendix Supplementary materials

Image, application 1
