# Supplementary material for: Family-centred care in adult intensive care units: A scoping review protocol of secondary evidence with a focus on the nursing role
Source: MethodsX. 2026 Sep 3;17:104142. doi: 10.1016/j.mex.2026.104142 (PMC13579151; doi:10.1016/j.mex.2026.104142)
Supplement: Supplementary file 1 [file mmc1.docx]

***Appendix 1. Master Source Extraction Form Matrix***

| **Variable Name** | **Field Label** | **Allowed Values / Coding** | **Extraction Rule** | **Required** |
| --- | --- | --- | --- | --- |
| source_id | Source ID | Unique alphanumeric code | Assign a unique ID to each included source | Yes |
| citation_full | Full citation | Free text | Enter full bibliographic citation | Yes |
| first_author | First author / org | Free text | Enter first author surname or issuing organization | Yes |
| publication_year | Publication year | YYYY | Record year of publication | Yes |
| title | Title | Free text | Enter full title | Yes |
| source_type | Type of source | Categorical | Systematic review; Meta-analysis; Scoping; Integrative; Narrative; Guideline; Consensus; Position | Yes |
| journal_or_publisher | Journal / publisher | Free text | Journal name or issuing body | Yes |
| country_of_origin | Country of origin | Free text | Country of first author or organization | Yes |
| language | Language | Categorical | English; Italian | Yes |
| peer_reviewed | Peer reviewed | Categorical | Yes; No; Unclear | Yes |
| objective | Objective | Free text | Extract stated review/guideline objective(s) | Yes |
| review_question_reported | RQ reported | Categorical | Yes; No | Yes |
| review_question_text | RQ text | Free text | Copy/summarize the stated review question(s) if present | No |
| search_period | Search period | Free text | Record search dates/range if reported | No |
| databases_searched | Databases | Semicolon-separated | Extract all databases or information sources searched | No |
| number_of_included_records | No. studies | Integer | Record total number synthesized in the source | No |
| critical_appraisal_conducted | Appraisal done | Categorical | Yes; No; Unclear | Yes |
| appraisal_tool | Appraisal tool | Text | AMSTAR; JBI; CASP; GRADE; Other; Not reported | No |
| meta_analysis_conducted | Meta-analysis done | Categorical | Yes; No | Yes |
| adult_icu_only | Adult ICU only | Categorical | Yes; No; Mixed but separable; Mixed not separable | Yes |
| icu_setting_types | ICU setting types | Free text | General ICU; Medical; Surgical; Cardiac; Neuro; Mixed; CCU; Other | Yes |
| populations_addressed | Populations | Semicolon-separated | Nurses; Patients; Family members/caregivers; Multidisciplinary staff; Mixed | Yes |
| nursing_focus_level | Nursing focus level | Categorical | Primary focus; Relevant but not central; Marginal; Not specific | Yes |
| nursing_data_extractable | Nursing data extractable | Categorical | Yes; No; Partially | Yes |
| family_definition_reported | Family def reported | Categorical | Yes; No | Yes |
| family_definition_text | Family definition text | Free text | Copy/summarize the definition used | No |
| fcc_term_used | Terminology used | Semicolon-separated | Family-centred care; Family engagement; Family involvement; Open visitation; Other | Yes |
| fcc_definition_reported | FCC def reported | Categorical | Yes; No | Yes |
| fcc_definition_text | FCC definition text | Free text | Copy/summarize the operational definition | No |
| conceptual_framework_reported | Framework reported | Categorical | Yes; No | Yes |
| conceptual_framework_name | Framework name | Free text | Record framework/model if present | No |
| nurse_role_described | Nursing role described | Categorical | Yes; No; Partially | Yes |
| nurse_role_summary | Summary of nursing role | Free text | Summarize role: mediator, facilitator, educator, gatekeeper, advocate, coordinator | No |
| nurse_family_relationship_described | Relationship described | Categorical | Yes; No; Partially | Yes |
| nurse_family_relationship_type | Relationship type | Categorical | Provider-led; Collaborative/partnership; Negotiated; Shared decision-making; Unclear | Yes |
| fcc_domains | FCC domains | Semicolon-separated | Communication; Presence; Support; Care participation; Decision-making; Environmental | Yes |
| implementation_barriers_reported | Barriers reported | Categorical | Yes; No | Yes |
| implementation_facilitators_reported | Facilitators reported | Categorical | Yes; No | Yes |
| instruments_reported | Instruments reported | Categorical | Yes; No | Yes |
| psychometric_info_reported | Psychometric info | Categorical | Yes; No; Limited | Yes |
| outcomes_reported | Outcomes reported | Categorical | Yes; No | Yes |
| key_findings | Key findings | Free text | Summarize main findings relevant to review questions | Yes |
| knowledge_gaps | Knowledge gaps | Free text | Extract reported conceptual, methodological, geographic, or measurement gaps | Yes |
| implications_for_nursing | Implications for nursing | Free text | Summarize implications for ICU nursing practice and nurse-family relationship | Yes |
| reviewer_notes | Reviewer notes | Free text | Record ambiguity, concerns, missing data, or coding decisions | No |

***Appendix 2. Intervention / Practice Model Extraction Form Matrix***

| **Variable Name** | **Field Label** | **Allowed Values / Coding** | **Extraction Rule** | **Required** |
| --- | --- | --- | --- | --- |
| intervention_id | Intervention ID | Unique alphanumeric | Unique record ID | Yes |
| source_id | Linked source ID | Must match master table | Link to parent source | Yes |
| intervention_name | Intervention name | Free text | Record name used in source | Yes |
| intervention_category | Category | Categorical | Open visiting; Presence during procedures; Structured meetings; Care participation; ICU diary; Emotional support | Yes |
| intervention_description | Description | Free text | Brief structured narrative summary | Yes |
| intervention_level | Level | Categorical | Bedside; Unit-level; Organizational; Health-system | Yes |
| intervention_target | Target population | Semicolon-separated | Family; Patient; Nurses; Multidisciplinary staff; Mixed | Yes |
| nurse_led | Nurse-led | Categorical | Yes; No; Partially; Unclear | Yes |
| multidisciplinary | Multidisciplinary | Categorical | Yes; No; Unclear | Yes |
| operationalization_details | Operationalization | Free text | Note frequency, duration, components, training, materials | Yes |
| implementation_context | Context details | Free text | ICU subtype, policy context, organizational context | No |
| reported_effect_direction | Effect direction | Categorical | Positive; Mixed; No clear effect; Negative/harm; Not assessed | No |

***Appendix 3. Barriers and Facilitators Extraction Form Matrix***

| **Variable Name** | **Field Label** | **Allowed Values / Coding** | **Extraction Rule** | **Required** |
| --- | --- | --- | --- | --- |
| implementation_item_id | Item ID | Unique alphanumeric | Unique record ID | Yes |
| source_id | Linked source ID | Must match master table | Link to parent source | Yes |
| item_type | Item type | Categorical | Barrier; Facilitator | Yes |
| item_label | Standardized label | Free text | Short standardized label | Yes |
| item_description | Description | Free text | Extract or summarize specific item description | Yes |
| implementation_level | Level | Categorical | Individual; Interpersonal/relational; Organizational; Environmental; Cultural; System | Yes |
| stakeholder_group | Stakeholder affected | Semicolon-separated | Nurses; Family members; Patients; Team; Organization; Mixed | No |
| example_keywords | Example keywords | Semicolon-separated | Workload; Staffing; ICU design; Training; Leadership; Culture; Time; Role ambiguity | No |
| geographic_or_cultural_context | Specific context | Free text | Record if explicitly linked to a localized geographic/cultural context | No |

***Appendix 4. Measurement Instrument Extraction Form Matrix***

| **Variable Name** | **Field Label** | **Allowed Values / Coding** | **Extraction Rule** | **Required** |
| --- | --- | --- | --- | --- |
| instrument_record_id | Instrument ID | Unique alphanumeric | Unique record ID | Yes |
| source_id | Linked source ID | Must match master table | Link to parent source | Yes |
| instrument_name | Instrument name | Free text | Record full name and abbreviation | Yes |
| construct_measured | Construct measured | Semicolon-separated | Family satisfaction; Family needs; Anxiety; Depression; PTSD; Communication quality; Staff attitudes | Yes |
| target_population | Target population | Categorical | Family members; Nurses; Patients; Multidisciplinary staff; Mixed | Yes |
| instrument_use_type | Use type | Categorical | Outcome measure; Screening tool; Satisfaction survey; Implementation evaluation; Validation | Yes |
| icu_specific | ICU-specific | Categorical | Yes; No; Unclear | Yes |
| validated_reported | Validation reported | Categorical | Code whether source reports validation status | Yes |
| psychometric_properties | Psychometric metrics | Semicolon-separated | Validity; Reliability; Internal consistency; Test-retest; Feasibility; Responsiveness | Yes |
| psychometric_summary | Psychometric summary | Free text | Summarize what the source concludes about psychometric quality | No |
| clinical_applicability_reported | Applicability reported | Categorical | Code whether real-world usability is discussed | Yes |
| applicability_summary | Applicability summary | Free text | Time burden, clinical feasibility, ease of use, language versions | No |

***Appendix 5. Outcome Extraction Form Matrix***

| **Variable Name** | **Field Label** | **Allowed Values / Coding** | **Extraction Rule** | **Required** |
| --- | --- | --- | --- | --- |
| outcome_record_id | Outcome ID | Unique alphanumeric | Unique record ID | Yes |
| source_id | Linked source ID | Must match master table | Link to parent source | Yes |
| outcome_group | Outcome group | Categorical | Family; Staff; Patient; Organizational | Yes |
| outcome_name | Outcome name | Free text | Record explicit outcome label used | Yes |
| outcome_definition | Operational definition | Free text | Extract definition if explicitly reported | No |
| outcome_measure | Outcome measure/tool | Free text | Record specific instrument or indicator if reported | No |
| outcome_direction | Direction of findings | Categorical | Improved; Worsened; No difference; Mixed; Inconclusive | Yes |
| effect_summary | Summary of findings | Free text | Narrative summary of statistical or qualitative effect | Yes |
| pooled_estimate_reported | Pooled estimate done | Categorical | Code whether pooled quantitative meta-analysis result exists | Yes |
| pooled_estimate_text | Pooled estimate text | Free text | Short summary of pooled effect sizes/confidence intervals if reported | No |
